# Mutations in the PP2A regulatory subunit B family genes *PPP2R5B*, *PPP2R5C* and *PPP2R5D* cause human overgrowth

**DOI:** 10.1093/hmg/ddv182

**Published:** 2015-05-13

**Authors:** Chey Loveday, Katrina Tatton-Brown, Matthew Clarke, Isaac Westwood, Anthony Renwick, Emma Ramsay, Andrea Nemeth, Jennifer Campbell, Shelagh Joss, McKinlay Gardner, Anna Zachariou, Anna Elliott, Elise Ruark, Rob van Montfort, The Childhood Overgrowth Collaboration, Nazneen Rahman

**Affiliations:** 1 Division of Genetics and Epidemiology,; 2 Cancer Research UK Cancer Therapeutics Unit and Division of Structural Biology, Institute of Cancer Research, London, UK,; 3 Medical Genetics Unit, St George's University of London, London, UK,; 4 Cancer Genetics Unit, Royal Marsden Hospital, London, UK,; 5 Department of Clinical Genetics, Churchill Hospital, Oxford, UK,; 6 Institute of Genetic Medicine, International Centre for Life, Newcastle University, Newcastle upon Tyne, UK,; 7 West of Scotland Genetic Services, Southern General Hospital, Scotland, UK,; 8 Yorkshire Regional Clinical Genetics Service, Chapel Allerton Hospital, Leeds, UK and; 9 Genetic Health Service New Zealand, Wellington Hospital, Wellington, NZ

## Abstract

Overgrowth syndromes comprise a group of heterogeneous disorders characterised by excessive growth parameters, often in association with intellectual disability. To identify new causes of human overgrowth, we have been undertaking trio-based exome sequencing studies in overgrowth patients and their unaffected parents. Prioritisation of functionally relevant genes with multiple unique *de novo* mutations revealed four mutations in protein phosphatase 2A (PP2A) regulatory subunit B family genes *protein phosphatase 2, regulatory Subunit B’, beta (PPP2R5B)*; *protein phosphatase 2, regulatory Subunit B’, gamma (PPP2R5C)*; and *protein phosphatase 2, regulatory Subunit B’, delta (PPP2R5D).* This observation in 3 related genes in 111 individuals with a similar phenotype is greatly in excess of the expected number, as determined from gene-specific *de novo* mutation rates (*P* = 1.43 × 10^−10^). Analysis of exome-sequencing data from a follow-up series of overgrowth probands identified a further pathogenic mutation, bringing the total number of affected individuals to 5. Heterozygotes shared similar phenotypic features including increased height, increased head circumference and intellectual disability. The mutations clustered within a region of nine amino acid residues in the aligned protein sequences (*P* = 1.6 × 10^−5^). We mapped the mutations onto the crystal structure of the PP2A holoenzyme complex to predict their molecular and functional consequences. These studies suggest that the mutations may affect substrate binding, thus perturbing the ability of PP2A to dephosphorylate particular protein substrates. PP2A is a major negative regulator of v-akt murine thymoma viral oncogene homolog 1 (AKT). Thus, our data further expand the list of genes encoding components of the phosphatidylinositol-4,5-bisphosphate 3-kinase (PI3K)/AKT signalling cascade that are disrupted in human overgrowth conditions.

## Introduction

Overgrowth syndromes are a heterogeneous group of disorders characterised by excess prenatal and postnatal growth compared with the age-matched peer group. While for the majority of individuals the molecular aetiology of their condition remains unknown, several overgrowth-associated genes have been identified in recent years, many of which encode components of the phosphatidylinositol-4,5-bisphosphate 3-kinase (PI3K)/v-akt murine thymoma viral oncogene homolog 1 (AKT) growth regulatory cascade (Supplementary Material, Fig. S1) (1).

*De novo* gene mutations are a cause of several overgrowth conditions, and their detection by exome sequencing has proved a successful strategy for the identification of novel genetic causes of overgrowth syndromes (2,3). To further leverage this strategy, we are undertaking trio-based exome sequencing in individuals with a range of overgrowth phenotypes and their unaffected parents. Herein, we report five affected individuals from unrelated families with *de novo* mutations in the protein phosphatase 2A (PP2A) regulatory subunit B family genes *protein phosphatase 2, regulatory Subunit B’, beta (PPP2R5B)*, *protein phosphatase 2, regulatory Subunit B’, gamma (PPP2R5C)* and *protein phosphatase 2, regulatory Subunit B’, delta (PPP2R5D)*.

## Results

**Figure 1.**
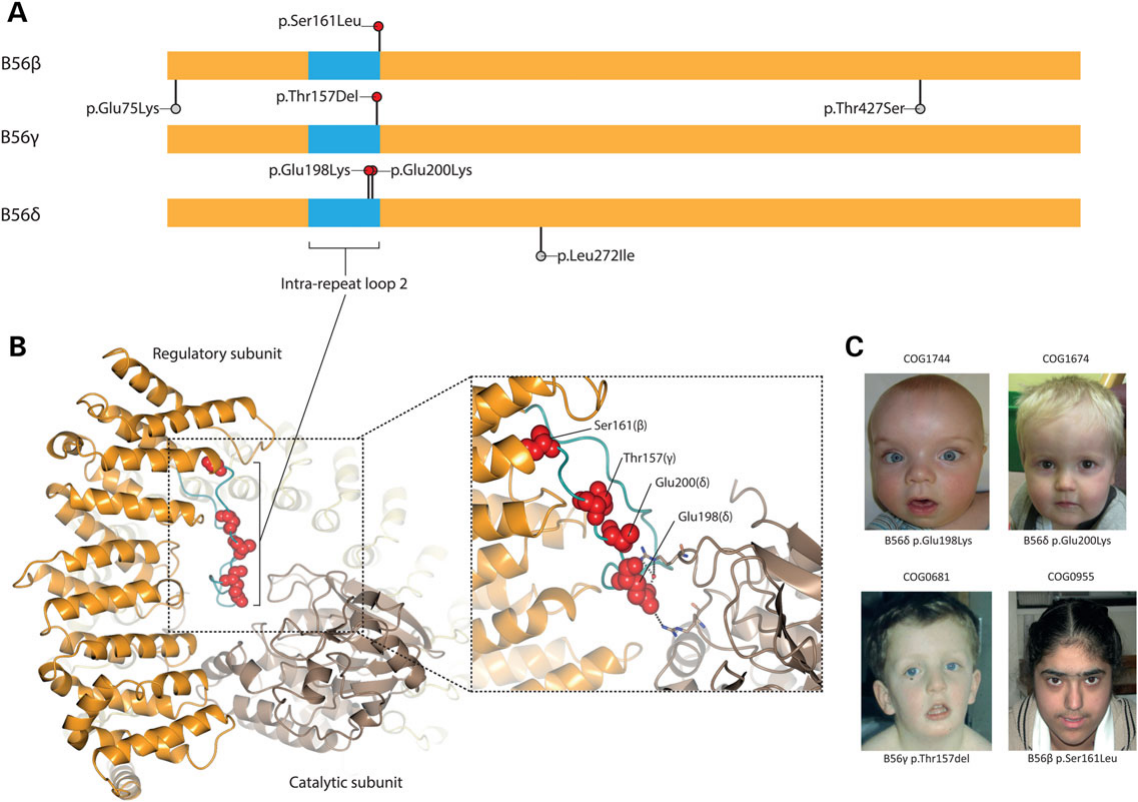
PP2A-B56 mutations in human overgrowth. (**A**) Protein schematic showing the distribution of PP2A-B56 subunit mutations in individuals with overgrowth (above, red lollipops) versus variants identified in the ICR1000 control series (below, grey lollipops). The shared core B56 domain is shown in orange and the intra-repeat loop 2 region in blue. (**B**) PP2A-B56 subunit mutations mapped onto the PP2A-B56γ complex. PP2A-B56γ is shown in orange, the catalytic subunit is in light brown and the scaffold subunit is in yellow. The intra-repeat loop 2 of PP2A-B56γ is highlighted in blue. The residues corresponding to the mutations identified are shown as red spheres. Inset: interaction of PP2A-B56γ p.Glu153 with the PP2A-Cα subunit. The glutamate side chain forms an ion pair interaction and water-mediated H-bonds to Arg residues from the catalytic subunit. (**C**) Facial characteristics of individuals with PP2A-B56 subunit mutations. Growth parameters and clinical features are described in Table 1. Specific consent to publish facial photographs was obtained for all individuals.

We have completed exome sequencing in 111 trios (Supplementary Material, Table S1) using Illumina exome enrichment kits and sequencers, performing alignment and variant calling with our in-house pipeline, identifying a total of 94 putative *de novo* coding variants. We first considered genes with more than one *de novo* mutation, amongst which was *PPP2R5D* (RefSeq accession number NM_006245), in which closely located *de novo* non-synonymous mutations, c.592G>A (p.Glu198Lys) and c.598G>A (p.Glu200Lys), were identified in two individuals (Fig. 1, Table 1). *PPP2R5D* encodes B56δ, one of the PP2A regulatory B subunits. PP2A-B56 is a key cellular serine-threonine phosphatase with roles in multiple pathways including negative regulation of the PI3K/AKT growth regulatory cascade [reviewed in (4)]. Given the established role of this pathway in growth regulation and overgrowth phenotypes, this gene was of immediate interest.

**Table 1.** PP2A-B56 mutations and associated phenotypes

Case ID	Gene	Mutation		Inheritance	Age/years	Ht/	HC/	Intellectual disability	Other clinical features
		Nucleotide	Amino acid			s.d.	s.d.		
COG1744	*PPP2R5D*	c.592G>A	p.Glu198Lys	*de novo*	0.3	2.3	3.6	Yes	Hypospadias
COG1674	*PPP2R5D*	c.598G>A	p.Glu200Lys	*de novo*	1.7	−1.4	3.8	Yes	
COG0328	*PPP2R5D*	c.598G>A	p.Glu200Lys	unknown	14	2	3.8	Yes	Parkinsonism
COG0681	*PPP2R5C*	c.468_470delAAC	p.Thr157del	*de novo*	9.3	1.6	3.3	Yes	Facial asymmetry, conductive hearing loss
COG0955	*PPP2R5B*	c.482C>T	p.Ser161Leu	*de novo*	17.5	3	0.3	Yes	Swelling of PIPJ

Mutation positions in *PPP2R5B*, *PPP2R5C* and *PPP2R5D* correspond to RefSeq sequences NM_006244, NM_001161725 and NM_006245, respectively.

Ht/s.d., height/standard deviation; HC/s.d., head circumference/standard deviation; PIPJ, proximal interphalangeal joint.

*PPP2R5D* is one of five genes encoding PP2A-B56 subunit proteins; the others are protein phosphatase 2, regulatory Subunit B’, alpha (*PPP2R5A*); *PPP2R5B*, *PPP2R5C* and *protein phosphatase 2, regulatory Subunit B’, epsilon* ***(****PPP2R5E)*, which encode B56α, B56β, B56γ and B56ε, respectively. Given our initial findings, we reviewed the exome data at the other PP2A-B56 subunit genes, which revealed *de novo* mutations in *PPP2R5B* (c.482C>T [p.Ser161Leu]; RefSeq accession number NM_006244) and *PPP2R5C* (c.468_470delAAC [p.Thr157del]; RefSeq accession number NM_001161725) in a further two individuals. Notably, these mutations were at similarly positioned residues to those identified in *PPP2R5D* (Fig. 1A). We confirmed all four mutations were present in the relevant proband and absent from their parents by Sanger sequencing (Supplementary Material, Fig. S2). Additionally, none of the mutations were present in 1000 population controls (the ICR1000 series) in which exome sequencing had been performed using the same laboratory and analytical pipelines (4/111 versus 0/1000; *P* = 0.0001). Furthermore, the identification of 4 *de novo* mutations in 3 closely related genes in 111 individuals with a similar phenotype is greatly in excess of the expected number, as determined from gene-specific *de novo* mutation rates (*P* = 1.43 × 10^−10^; see Materials and Methods for test description).

We next analysed exome data at *PPP2R5B*, *PPP2R5C* and *PPP2R5D* in a separate series of 152 individuals with overgrowth phenotypes for whom parental DNA is not available (the singleton series; Supplementary Material, Table S1). We identified one individual with *PPP2R5D* p.Glu200Lys, which is likely causative of their phenotype as the identical mutation had occurred *de novo* in one of the trio series (Table 1). Of note, none of the five pathogenic mutations showed any evidence of mosaicism, either by the mutation read percentages in the next generation sequencing data or the Sanger sequencing traces (Supplementary Material, Fig. S2). Three other rare non-synonymous variants were detected, but in the absence of additional evidence, a causal relationship with overgrowth cannot be inferred for these variants (Supplementary Material, Table S2).

We undertook structural analysis to gain insights into the possible impact of the mutations we identified in overgrowth cases on PP2A-B56 function. The mutations were all located within a highly conserved region of the PP2A-B56 subunits; indeed, they clustered within a nine amino acid region in intra-repeat loop 2 (*P* = 1.6 × 10^−5^; Fig. 1A; Fig. 1B). PP2A exists as a heterotrimeric holoenzyme comprising a catalytic ‘C’, structural ‘A’ and a regulatory ‘B’ subunit. The crystallised structure of the B56 core domain comprises 18 alpha helices organised into 8 huntingtin, elongation factor 3 (EF3), protein phosphatase 2A (PP2A) and the yeast kinase TOR1 (HEAT)-like repeats that form an exposed concave surface residing adjacent to the catalytic site of the PP2A catalytic subunit (Fig. 1B). This concave surface is enriched with negatively charged amino acids and forms, along with the C subunit, a cleft for substrate binding. All identified mutations map to residues forming intra-repeat loop 2, which is also termed the substrate specificity loop and is located within the exposed substrate-binding cleft (Fig. 1B). The substrate specificity loop is believed to regulate both substrate recognition and orientation, such that the amino acid targeted for dephosphorylation is placed near the active site of the catalytic subunit (5). Thus, it is plausible that the mutations reported here cause impaired substrate-specific phosphatase activity owing to perturbed substrate recognition and/or positioning. Functional studies are required to confirm this hypothesis and to identify the targeted substrate protein(s).

We next evaluated the phenotypic data for individuals with pathogenic PP2A-B56 subunit mutations (Table 1, Supplementary Material). The facial features were not distinctive (Fig. 1C). The median head circumference was 3.6 standard deviations above the mean. Two of the three individuals with *PPP2R5D* mutations were also tall with heights greater than two standard deviations above the mean and thus had global overgrowth. In contrast, the individual with a *PPP2R5B* mutation had a normal head circumference but was very tall with a height three standard deviations above the mean (Table 1). All five individuals were said to have an intellectual disability by the referring clinician. These data indicate that overgrowth and intellectual disability are key clinical features of PP2A-B56 subunit mutations. However, it should be noted that increased height and/or head circumference is an eligibility criterion for the Childhood Overgrowth (COG) study together with at least one other clinical feature, which is often intellectual disability. Therefore, the full phenotypic spectrum of PP2A-B56 subunit mutations may be broader than demonstrated here. Moreover, the absence of distinctive clinical features associated with PP2A-B56 mutations further underscores the high genetic heterogeneity of overgrowth conditions. It is interesting that contemporaneously and independently of our study the Deciphering Developmental Disorders study identified four *de novo PPP2R5D* mutations amongst 1133 individuals with developmental disorders (6). Three had c.592G>A (p.Glu198Lys), the mutation we also identified and the other had c.602C>G (p.Pro201Arg), which is also located in the substrate specificity loop. Variable phenotypic features were observed, but one individual was noted to have increased head circumference (Supplementary Material, Table S3). To our knowledge, *de novo* mutations in *PPP2R5B* and *PPP2R5C* have not been reported.

## Discussion

We have identified heterozygous missense mutations in PP2A regulatory subunit B family genes *PPP2R5B*, *PPP2R5C* and *PPP2R5D* in individuals with overgrowth and intellectual disability. Structural analysis indicates that the mutations affect the PP2A-B56 substrate specificity loop, plausibly altering the ability of PP2A-B56 to dephosphorylate target substrates.

PP2A has been implicated in a multitude of signalling cascades that control cell proliferation, division, differentiation, apoptosis, metabolism, adhesion and migration and is thought to modulate the activity of over 30 different kinases [reviewed in (4)]. This diversity in PP2A function is conferred in part by the range of different regulatory B subunits, with each B subunit family directing the PP2A complex to distinct and overlapping targets. Multiple lines of evidence indicate that the PP2A-B56 holoenzyme has particular specificity for AKT, a major component of the PI3K/AKT growth regulatory cascade. More specifically, PP2A-B56 has been shown to inhibit AKT activity in specific subcellular compartments through the direct dephosphorylation of AKT Thr308 and Ser473 (7,8). In its phosphorylated state, AKT promotes cellular growth and proliferation. Thus, in normal cells, PP2A-B56 is a negative regulator of the PI3K/AKT growth regulatory cascade.

It is plausible that the mutations described here disrupt the ability of PP2A-B56 to dephosphorylate AKT, thus disrupting its ability to negatively regulate the PI3K/AKT growth regulatory cascade. Indeed, multiple genes encoding components of the PI3K/AKT growth regulatory cascade have been causally implicated in human growth disorders (Supplementary Material, Fig. S1). Foremost amongst these is *phosphatase and tensin holog (PTEN)*, inactivating mutations of which cause increased head circumference, a core feature of all the *PTEN*-associated syndromes, such as Bannayan Riley Ruvalcaba syndrome (MIM 153480) and Cowden syndrome (MIM 158350) (9). In addition, somatic activating *phosphatidylinosiltol-4,5-bisphosphate 3-kinase, catalytic subunit alpha (PIK3CA)* mutations and germline activating *phosphoinositide-3-kinase, regulatory subunit 2 (beta) (PIK3R2)* mutations (both encoding PI3K regulatory subunits) cause conditions characterized by increased head circumference; megalencephaly-capillary malformation (MCAP [MIM 602501]) and megalencephaly-polymicrogyria-polydactyly-hydrocephalus (MPPH [MIM 603387]) syndromes, respectively (10). Activating germline and somatic *AKT3* mutations has also been described in a small number of individuals with atypical MPPH and hemimegalencephaly (MPPH2 [MIM 615937]) (10). Finally, activating somatic mutations in *AKT1* cause Proteus syndrome (MIM 176920), a highly variable disorder of asymmetric overgrowth, tissue hyperplasia affecting multiple organs and increased tumour risk (Supplementary Material, Fig. S1) (11). These findings, in conjunction with the data presented here, suggest that further components of the PI3K/AKT pathway may also be involved in human growth disorders.

## Materials and Methods

### Case series

We recruited individuals with overgrowth through the COG study. The research was approved by the London Multicentre Ethics Committee (reference MREC/01/2/44), and informed consent was obtained from all participants and/or families. We extracted DNA from peripheral blood. We obtained phenotypic information through a standardized questionnaire. A summary of the overgrowth phenotypes used in this study is given in Supplementary Material, Table S1. A full list of collaborators is given in the Supplementary Material.

### ICR1000 control series

We used lymphocyte DNA from 1000 population-based controls obtained from the 1958 Birth Cohort Collection (http://www2.le.ac.uk/projects/birthcohort), a continuing follow-up study of individuals born in the UK in 1 week in 1958. The ICR1000 series [Accession number: EGAS00001000971] is accessible from the European Genome-phenome Archive (https://www.ebi.ac.uk/ega/datasets/EGAD00001001021).

### Exome sequencing

We performed target enrichment using either the TruSeq Exome Enrichment Kit or the Nextera Exome Enrichment Kit, targeting 62 Mb and 37 Mb of the human genome, respectively. Captured DNA libraries were PCR amplified using the supplied paired-end PCR primers. Sequencing was performed on either an Illumina HiSeq 2000 or 2500, generating 2 × 101-bp and 2 × 100-bp reads, respectively.

### Exome variant calling and *de novo* mutation detection

We used Stampy (version 1.0.14) to map sequencing reads to the human reference genome (hg19). Duplicate reads were flagged using Picard version 1.60. We used Platypus version 0.1.5 to perform variant calling whereas annotation was performed by CAVA, a variant annotation tool written in Python that follows Human Genome Variation Society nomenclature and ensures consistent annotation of indels (http://www.well.ox.ac.uk/cava).

Prior to the *de novo* analyses, we checked the pairwise relatedness of all individuals in each trio, confirming biological parentage in all. For the *de novo* analyses, we used a custom script written in Perl to remove variants that were either present or not sufficiently covered in the corresponding parental samples or were supported by less than three reads. We then prioritised genes for follow-up based on number of *de novo* mutations and candidacy. Following the identification of *de novo* mutations in *PPP2R5B, PPP2R5C* and *PPP2R5D* in the trio series, we reviewed all high-quality variant calls in these genes in the singleton series and the ICR1000 control series.

### Sanger sequencing

We used Sanger sequencing of M13 tagged-PCR products from genomic DNA to validate all variants identified in probands by exome sequencing and to confirm the absence in parental samples as appropriate. Primer hybridisation sequences used for PCR are given in Supplementary Material, Table S4. PCRs were performed using the Qiagen Multiplex PCR Kit according to the manufacturer's instructions. PCR products were sequenced using M13 sequencing primers, the BigDye Terminator Cycle Sequencing Kit and an ABI 3730 Genetic Analyser (Applied Biosystems). Sequences were analysed using Mutation Surveyor software v3.20 (SoftGenetics) and verified by manual inspection.

### Genbank accession numbers

Mutation positions were assigned according to the following human mRNA reference sequences: NM_006244 (*PPP2R5B*), NM_001161725 (*PPP2R5C*) and NM_006245 (*PPP2R5D*).

### Statistical analyses

We compared mutation frequencies using a two-sided Fisher's Exact test. The significance of the observed pattern of multiple *de novo* mutations across the genes *PPP2R5B*, *PPP2R5C* and *PPP2R5D* was assessed using the method described in (8). Briefly, we calculated the number of expected *de novo* mutations across the three genes by summing the gene-specific mutation rates [as defined by Samocha *et al*. (12)] by the number of transmissions (i.e. twice the number of probands). This value was then used as the mean of a Poisson distribution from which the probability of getting the observed number of mutations (i.e. four) within these genes was taken.

We undertook clustering analysis to assess the significance of the four *de novo* mutations occurring within a region of nine amino acid residues in the aligned protein sequences. Using one million permutations, we randomly placed mutations within each of the three genes according to the number observed, calculating the distance in bases between the two furthest mutations. The proportion of simulated mutations occurring within nine amino acids or fewer was compared with the observed number to determine the *P*-value.

### Structural analyses

PP2A-B56 variants were mapped to their corresponding residues in the crystal structure of the PP2A holoenzyme (Aα-B56γ-Cα) in complex with Shugoshin-like 1 (Sgo1) (Protein Data Bank accession code 3FGA) (13). Images were generated with CCP4MG (14). The shugoshin Sgo1 homodimer and inhibitor toxin microcystin peptide have been omitted for clarity.

## Supplementary Materials

Supplementary Material is available at *HMG* online.

## Funding

This work was supported by Wellcome Trust Award 100210/Z/12/Z.I.W. and R.v.M. are supported by Cancer Research UK program grant C309/A11566 and The Institute of Cancer Research. Access to these resources was enabled via the 58READIE Project funded by Wellcome Trust and Medical Research Council (grant numbers WT095219MA and G1001799). N.R. acknowledges support from the NIHR RM/ICR Biomedical Research Centre. Funding to pay the Open Access publication charges for this article was provided by Wellcome Trust.

## Supplementary Material

Supplementary Data
