# Corrigendum: Mutations in the PP2A regulatory subunit B family genes *PPP2R5B*, *PPP2R5C* and *PPP2R5D* cause human overgrowth

**DOI:** 10.1093/hmg/ddy424

**Published:** 2019-01-07

**Authors:** Chey Loveday, Katrina Tatton-Brown, Matthew Clarke, Isaac Westwood, Anthony Renwick, Emma Ramsay, Andrea Nemeth, Jennifer Campbell, Shelagh Joss, McKinlay Gardner, Anna Zachariou, Anna Elliott, Elise Ruark, Rob van Montfort, The Childhood Overgrowth Collaboration, Nazneen Rahman

**Affiliations:** 1 Division of Genetics and Epidemiology; 2 Cancer Research UK Cancer Therapeutics Unit and Division of Structural Biology, Institute of Cancer Research, London, UK; 3 Medical Genetics Unit, St George’s University of London, London, UK; 4 Cancer Genetics Unit, Royal Marsden Hospital, London, UK; 5 Department of Clinical Genetics, Churchill Hospital, Oxford, UK; 6 Institute of Genetic Medicine, International Centre for Life, Newcastle University, Newcastle upon Tyne, UK; 7 West of Scotland Genetic Services, Southern General Hospital, Scotland, UK; 8 Yorkshire Regional Clinical Genetics Service, Chapel Allerton Hospital, Leeds, UK; 9 Genetic Health Service New Zealand, Wellington Hospital, Wellington, NZ

The authors wish to apologize for the following error: the name of M. Tischkowitz was spelled incorrectly in the supplementary material. This has been corrected online.

## Supplementary Material

ddv182supp_ddy424

