# Supplementary material for: Mutations in the PP2A regulatory subunit B family genes PPP2R5B, PPP2R5C and PPP2R5D cause human overgrowth
Source: Hum Mol Genet. 2015 May 13;24(17):4775–9. doi: 10.1093/hmg/ddv182 (PMC4527483; doi:10.1093/hmg/ddv182)
Supplement: Supplementary Data [file supp_24_17_4775__index.html]

Mutations in the PP2A regulatory subunit B family genes PPP2R5B, PPP2R5C and PPP2R5D cause human overgrowth — Mutations in the PP2A regulatory subunit B family genes PPP2R5B, PPP2R5C and PPP2R5D cause human overgrowth — Mutations in the PP2A regulatory subunit B family genes PPP2R5B, PPP2R5C and PPP2R5D cause human overgrowth — Mutations in the PP2A regulatory subunit B family genes PPP2R5B, PPP2R5C and PPP2R5D cause human overgrowth — Supplementary Data 

# Mutations in the PP2A regulatory subunit B family genes *PPP2R5B*, *PPP2R5C* and *PPP2R5D* cause human overgrowth

## Supplementary Data

Supplementary Data

- Supplementary Data - Docx file
