# Supplementary material for: Mutations in the PP2A regulatory subunit B family genes PPP2R5B, PPP2R5C and PPP2R5D cause human overgrowth
Source: Hum Mol Genet. 2015 May 13;24(17):4775–9. doi: 10.1093/hmg/ddv182 (PMC4527483; doi:10.1093/hmg/ddv182)
Supplement: Supplementary Data [file supp_ddv182_ddv182supp.docx]

**Supplementary Material**

**Mutations in the PP2A regulatory subunit B family genes *PPP2R5B*, *PPP2R5C* and *PPP2R5D* cause human overgrowth and intellectual disability**

Chey Loveday^1^[†](http://hmg.oxfordjournals.org/content/24/1/230.full#fn-1), Katrina Tatton-Brown^1,2,3^[†](http://hmg.oxfordjournals.org/content/24/1/230.full#fn-1)*, Matthew Clarke^1^, Isaac Westwood^4^, Anthony Renwick^1^, Emma Ramsay^1^, Andrea Nemeth^5^, Jennifer Campbell^6^, Shelagh Joss^7,8^, McKinlay Gardner^9^, Anna Zachariou^1^, Anna Elliott^1^, Elise Ruark^1^, Rob van Montfort^4^, The Childhood Overgrowth Collaboration^1^, Nazneen Rahman^1,3^*

^1^Division of Genetics and Epidemiology, Institute of Cancer Research, London, UK

^2^Medical Genetics Unit, St George’s University of London, London, UK

^3^Cancer Genetics Unit, Royal Marsden Hospital, London, UK

^4^Cancer Research UK Cancer Therapeutics Unit and Division of Structural Biology, Institute of Cancer Research, London, UK

^5^Department of Clinical Genetics, Churchill Hospital, Oxford, UK

^6^Institute of Genetic Medicine, International Centre for Life, Newcastle University, UK

^7^West of Scotland Genetic Services, Southern General Hospital, Scotland, UK

^8^Yorkshire Regional Clinical Genetics Service, Chapel Allerton Hospital, Leeds, UK

^9^Genetic Health Service New Zealand, Wellington Hospital, NZ

*To whom correspondence should be addressed. NR, Tel: +44 20 8722 4145; Fax: +44 20 8722 4359; email:[rahmanlab@icr.ac.uk](mailto:rahmanlab@icr.ac.uk).

[†](http://hmg.oxfordjournals.org/content/24/1/230.full#fn-1)These authors contributed equally to the work.

**Table S1**. Summary of Overgrowth series

| **Growth phenotypes** | **Trio series** | **Singleton series** | **Total** |
| --- | --- | --- | --- |
| Overgrowth (Ht and HC > 2SD) | 50 | 52 | 102 |
| Isolated macrocephaly (HC >2SD) | 21 | 43 | 64 |
| Isolated tall stature (ht >2SD) | 35 | 41 | 76 |
| Other* | 5 | 16 | 21 |
| Total | 111 | 152 | 263 |

Ht = height, HC = Head circumference. *Only one growth parameter was available, and therefore could not be classified into one of the other groups.

**Table S2**. PP2A-B56 variants in overgrowth cases and controls

| **Gene** | **Mutation** | | **Overgrowth Series** | | **ICR1000** | **ExAC** | **Polyphen** | **SIFT** |
| --- | --- | --- | --- | --- | --- | --- | --- | --- |
|  | **Nucleotide** | **Amino acid** | **Trios** | **Singleton** |  |  |  |  |
| PPP2R5B | c.110G>A | p.Arg37His | 1 | 0 | 0 | 5 | Probably Damaging | Deleterious |
| PPP2R5B | c.223G>A | p.Glu75Lys | 0 | 0 | 2 | 1 | Possibly Damaging | Tolerated |
| **PPP2R5B** | **c.482C>T** | **p.Ser161Leu** | **1*** | **0** | **0** | **1** | **Probably Damaging** | **Tolerated** |
| PPP2R5B | c.528G>T | p.Leu176Phe | 0 | 0 | 1 | 0 | Probably Damaging | Deleterious |
| PPP2R5B | c.884A>G | p.His295Arg | 0 | 1 | 0 | 0 | Probably Damaging | Deleterious |
| PPP2R5B | c.1280C>G | p.Thr427Ser | 0 | 0 | 1 | 6 | Benign | Tolerated |
| **PPP2R5C** | **c.468_470delAAC** | **p.Thr157del** | **1*** | **0** | **0** | 0 |  |  |
| PPP2R5C | c.622C>T | p.Arg208Trp | 0 | 1 | 0 | 0 | Probably Damaging | Tolerated |
| PPP2R5C | c.1636G>C | p.Ala546Pro | 10 | 15 | 40 | 12088 | Benign | Tolerated |
| PPP2R5D | c.188C>T | p.Thr63Met | 0 | 1 | 0 | 3 | Possibly Damaging | Deleterious |
| **PPP2R5D** | **c.592G>A** | **p.Glu198Lys** | **1*** | **0** | **0** | **0** | **Probably Damaging** | **Deleterious** |
| **PPP2R5D** | **c.598G>A** | **p.Glu200Lys** | **1*** | **1** | **0** | **0** | **Probably Damaging** | **Deleterious** |
| PPP2R5D | c.814C>A | p.Leu272Ile | 0 | 0 | 1 | 0 | Probably Damaging | Tolerated |
| PPP2R5D | c.1789G>A | p.Ala597Thr | 0 | 0 | 1 | 0 | Benign | Tolerated |

*confirmed *de novo*. Pathogenic mutations are in bold. Mutation positions in *PPP2R5B*, *PPP2R5C* and *PPP2R5D* correspond to RefSeq sequences NM_006244, NM_001161725 and NM_006245, respectively.

**Table S3.** *PPP2R5D* mutations and reported phenotypes (DDD study, 2014)

| **DECIPHER ID** | **Mutation** | | **Phenotypes** |
| --- | --- | --- | --- |
|  | **Nucleotide** | **Amino acid** |  |
| 257984 | c.592G>A | p.Glu198Lys | Intellectual disability severe, hydrocephalus, chronic diarrhea, hypoglycemia |
| 260011 | c.592G>A | p.Glu198Lys | Global developmental delay, seizures, ventriculomegaly, narrow forehead, downslanted palpebral fissures, pyloric stenosis, narrow palate ,generalized hypotonia, macrocephaly, ventriculomegaly |
| 262688 | c.602C>G | p.Pro201Arg | Global developmental delay, congenital muscular torticollis, congenital hip dislocation |
| 263463 | c.592G>A | p.Glu198Lys | Global developmental delay, deeply set eye, abnormality of vision, myopia, strabismus, generalized hypotonia |

Mutation positions in *PPP2R5B*, *PPP2R5C* and *PPP2R5D* correspond to RefSeq sequences NM_006244, NM_001161725 and NM_006245, respectively.

**Table S4.** Primer hybridisation sequences for Sanger validations

| **Gene** | **Nucleotide** | **Protein** | **Forward sequence (5’-3’)** | **Reverse Sequence (5’-3’)** |
| --- | --- | --- | --- | --- |
| *PPP2R5B* | c.110G>A | p.Arg37His | AGTCTGTCCAGTCTCACCCA | ACTCTCTCTCTTGGCAGCTG |
| *PPP2R5B* | c.223G>A | p.Glu75Lys | CCAGTGTGGGGTGATGTTTG | ACAGGGTTATTCTCCACGCA |
| *PPP2R5B* | c.482C>T | p.Ser161Leu | GTCGAGATTGTACCACTGCG | ACATATCTCTTGGCCACGGA |
| *PPP2R5B* | c.528G>T | p.Leu176Phe | AATGAGTGGGTGAGGCTGTT | CTTCCATCCTATCCCTGGCC |
| *PPP2R5B* | c.884A>G | p.His295Arg | AGGATTCTGGAAGGAGGGAC | TGGCTTCACAGAGCTGATATCT |
| *PPP2R5C* | c.468_470delAAC | p.Thr157del | GGGAAGCGGCTACTGTTAGA | TTCAACGGCCATCAACACAG |
| *PPP2R5C* | c.622C>T | p.Arg208Trp | AGAGTCTCCAGATTTCCAACCT | CAGAGGAGACGATGGGGTAG |
| *PPP2R5C* | c.1544A>G | p.Lys515Arg | ACACAGGTCCTCAAACGAGT | GGAAAAGAACTGGCGGGC |
| *PPP2R5D* | c.188C>T | p.Thr63Met | GTGGGAGGCATATCTTGGGA | CCTGTGACCAATTGCCAAGT |
| *PPP2R5D* | c.592G>A/c.598G>A | p.Glu198Lys/ p.Glu200Lys | TGGAGCTCTAACTGGCCCTA | AAAGAGAGGGCAAGGACAGG |
| *PPP2R5D* | c.814C>A | p.Leu272Ile | CTTTCCCTCCCTTGTACCCC | CCACCTCAGTAGCAGAAGGT |
| *PPP2R5D* | c.1789G>A | p.Ala597Thr | CCATTCCTCACCTTGTCCCT | GTAGGAACGTGAGGGGTCAG |

**Figure S1**. Core PI3K/AKT pathway proteins and associated overgrowth phenotypes.


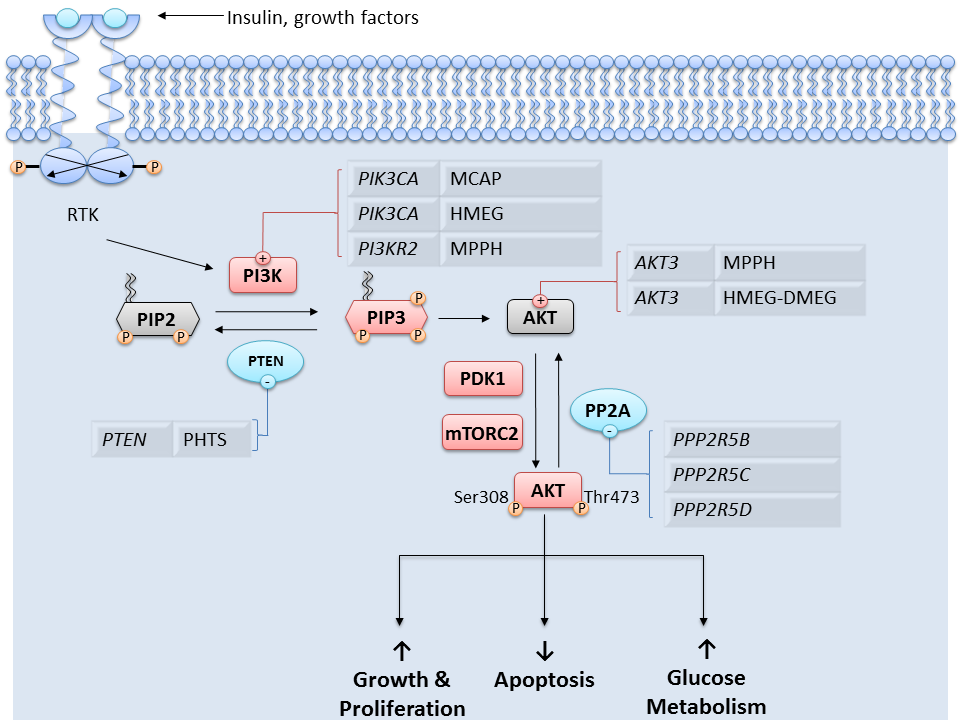
­­

­A group of related disorders termed the PTEN harmatoma tumour syndrome (PHTS), characterized by macrocephaly is caused by germline inactivating *PTEN* mutations. Megalencephaly-capilliary-malformation-polymicrogyria syndrome (MCAP) is predominantly caused by somatic activating *PIK3CA* mutations. Somatic activating *PIK3CA* mutations are also associated with hemimegalencephaly (HMEG). Megalencephaly-polymicrogyria-polydactyly-hydrocephalus syndrome (MPPH) is caused by germline activating *PI3KR2* mutations. Germline *AKT3* activating mutations have also been identified patients with atypical MPPH. Somatic activating *AKT3* mutations cause HMEG and dysplastic megalencephaly (DMEG). Finally, germline mutations in PP2A-B56 regulatory subunit B family genes *PPP2R5B*, *PPP2R5C* and *PPP2R5D* cause a novel overgrowth syndrome and intellectual disability.

**Figure S2**. Sanger sequencing electropherograms for *PPP2R5B*, *PPP2R5C* and *PPP2R5D* pathogenic mutations.

|  | **COG1744** | **COG1674** | **COG0328** | **COG0681** | **COG0955** |
| --- | --- | --- | --- | --- | --- |
| **Proband** | 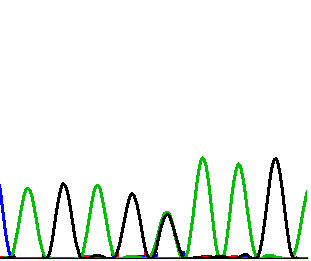  *PPP2R5D*  c.592G>A_ p.Glu198Lys | 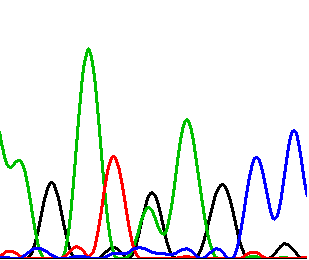  *PPP2R5D*  c.598G>A _ p.Glu200Lys | 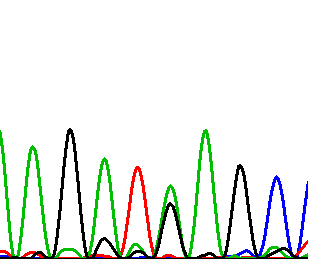  *PPP2R5D*  c.598G>A _ p.Glu200Lys | 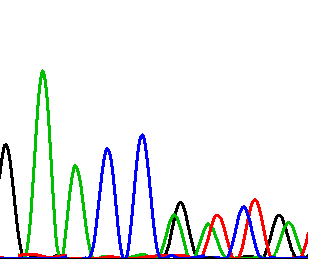  *PPP2R5C*  c.468_470delAAC_ p.Thr157del | 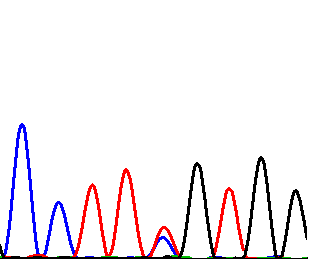  *PPP2R5B*  c.482C>T _ p.Ser161Leu |
| **Mum** | 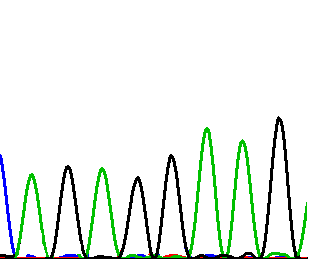 | 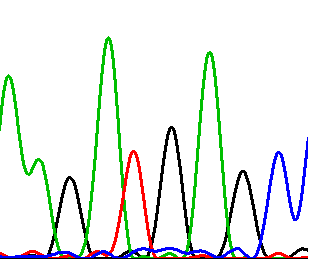 | **NA** | 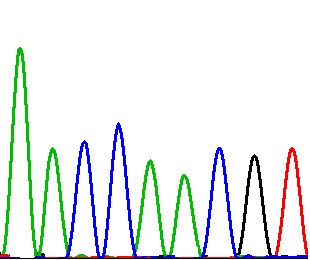 | 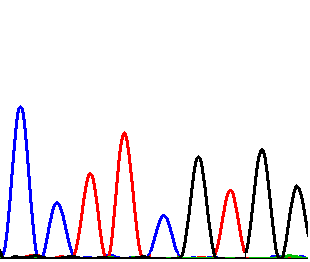 |
| **Dad** | 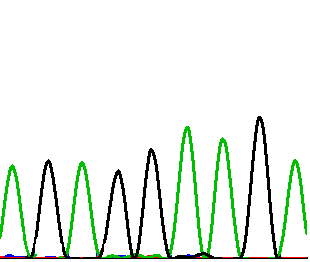 | 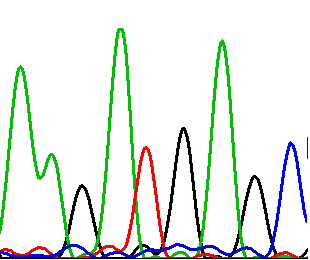 | **NA** | 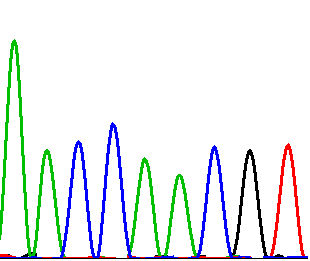 | 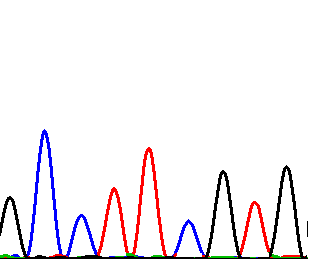 |

**Case Histories**

**COG1744** was born at term to healthy unrelated parents and following a normal pregnancy. His birth weight was 4300g (1.5SD), his birth length was 57cm (3SD) and birth head circumference was 38.5cm (2.6SD). In the immediate neonatal period, he had poor feeding and was subsequently diagnosed with pneumonia. In addition, he was noted to have a hypospadias with the meatus at the glandular-shaft junction. At the age of 9 months he was noted to be behind in his developmental milestones with poor head control and limited interaction. Genetic testing in the diagnostic laboratories included normal glypican 3 and array CGH analyses.

On examination of the proband at 3 months of age, height was 66cm (2.3SD), head circumference was 45.7cm (3.6SD) and weight was 7.65kg (1.8SD). He had a telecanthus, long palpebral fissures, pencilled eyebrows and a broad, high forehead with associated plagiocephaly (Figure **). In addition, he had deep palmar and plantar creases.

**COG1674** was born at 36 weeks following an uncomplicated pregnancy but labour was induced because of emeresis gravidarum. Postpartum he required IPPV and oxygen and developed jaundice, for which he required phototherapy, in the neonatal period. Data were not available for early growth parameters but by 20 months of age the proband was noted to have an isolated macrocephaly with a head circumference of 54.5cm (+3.8SD), height of 79.5cm (-1.4SD) and weight of 12.85kg (1.2SD). He was assessed as having a moderate intellectual disability but no other associated medical issues. The proband was not dysmorphic other than a broad, tall forehead and diagnostic investigations, undertaken by the referring clinician, included normal PTEN, maternal UPD14 and array CGH analyses. The family history was non-contributory.

**COG0328** was born at term following an uncomplicated pregnancy weighing 3600g (0.4SD). In early childhood she was noted to be hypotonic and had delayed motor milestones: she was crawling at approximately 15 months and walking at two years. By the age of 18 months her head circumference had increased to 52cm (3.4SD) and was 60cm by the age of 14 years (3.8SD). She was also tall with a height of 173cm at 14 years (2SD). She had a mild intellectual disability and, in her late twenties, developed an asymmetric resting tremor which progressed to the more florid features of dopa responsive Parkinsonism. There was no significant family history.

**COG0681** was born at 37 weeks gestation weighing 3100g (0.8SD) with a head circumference of 36cm (2.4SD). He was floppy with poor feeding in the neonatal period. He developed febrile seizures at the age of two years and was noted to have a moderate intellectual disability. There was no significant family history of note.

On examination he had a high arched palate, telecanthus and mild facial asymmetry.

**COG0955** was born at term to healthy, consanguineous parents. Her birth weight was 2948g (-1SD). She had no medical complications in the neonatal period but later in childhood, developed bilateral swelling of the proximal interphalangeal joints. She had a moderate intellectual disability and attended a school for special educational needs. Facially, COG0955 was noted to have synophrys and a low frontal hairline.

The proband had no significant family history and all five of her siblings had normal learning and growth.

**The Childhood Overgrowth (COG) Collaboration**

The following individuals coordinated recruitment and collection of the families and samples.

M. Ahmed, K. Anderson, K, H. Archer, R. Armstrong, D. Baralle, A. Barnicoat, M. Barrow, J. Barwell, P. Beales, K. Becker, J. Berg, B. Bernhard, S. Bhal, J. Birch, M. Bitner, E. Blair, M. Blyth, L. Bradley, A. Brady, G. Brice, L. Brueton, A. Burke, J. Burn, J. Campbell, N. Canham, B. Castle, K. Chandler, R. Chandrasena, C. Chu, D. Cilliers, A. Clarke, J. Clayton-Smith, V. Clowes, T. Cole, A. Colley, A. Collins, F. Connell, J. Cook, H. Cox, Y. Crow, T. Dabir, A. Dalton, R. Davidson, S. Davies, R. Day, D. Dearnaley, N. Dennis, C. Deshpande, B. Desouza, L. Devlin, A-M. Differ, R. Dinwiddie, A. Dixit, A. Dobbie, A. Donaldson, D. Donnai, D. Eastwood, I. Ellis, F. Elmslie, R. Evans, H. Firth, R. Fisher, D. Fitzpatrick, A. Flanagan, F. Flinter, P. Foley, N. Foulds, A. Fryer, A. Gallagher, S. Garcia, C. Gardiner, C. Garrett, M. Gerrard, R. Gibbons, D. Goudie, C. Graham, J. Hale, J. Harper, R. Harrison, H. Hughes, A. Henderson, P. Henman, R. Hennekam, E. Hobson, S. Hodgson, M. Holder, S. Holder, T. Homfray, Z. Huma, J. Hurst, M. Irving, L. Izatt, L. Jenkins, C. Jessen, D. Johnson, E. Jones, L. Jones, D. Josifova, S. Joss, Dr. Kanabar, B. Kerr, H. Kingston, J. Kingston, U. Kini, E. Kinning, A. Kumar, D. Kumar, K. Lachlan, W. Lam, M. Lees, G. Levitt, I. Lewis, A. Livesey, C. Longman, A. Lucassen, P. Lunt, J. MacDonnell, A. Magee, E. Maher, A. Male, S. Mansour, V. McConnell, M. McEntagart, R. McGowan, S. McKee, C. McKeown, C. Meany, S. Mehta, K. Metcalfe, S. Mohammed, G. Monaghan, T. Montgomery, A. Morgan, B. Morland, P. Morrison , J. Morton, R. Mudgal, A. Munaza, V. Murday, A. Nemeth, R. Newbury-Ecob, C. Oley, C. Owen, S-M. Park, M. Parker, C. Patel, M. Patton, S. Payne, D. Pilz, M. Pinkney, M. Pocha, C. Pottinger, K. Prescott, S. Price, K. Pritchard-Jones, A. Proctor, O. Quarrell, J. Rankin, L. Raymond, G. Rea, E. Reid, H.Rees, M. Robards, A. Roposch, E. Rosser, D. Rourke, D. Ruddy, A. Saggar, J. Sampson, R. Sandford, A. Sarkar, R. Scott, I. Scurr, R. Semple, S. Sharif, A. Shaw, C. Shaw-Smith, D. Shears, J. Shelagh, G. Smith, S. Smithson, M. Splitt, M. Stevens, A. Stewart, F. Stewart, H. Stewart, K. Stopps, M. Suri, E. Sweeney, G. Tanateles, A. Taylor, C. Taylor, K. Temple, E. Thomas, M. Tischowitz, J. Tolmie, S. Tomkins, P. Turnpenny, M. Van-Haelst, P. Vasudevan, E. Wakeling, L. Walker, D. Williams, L. Wilson, R. Winter, G. Woods, M. Wright, M-C. Addor, M. Akgul, D. Amor, R. Anderson, S. Andries, P. Ashton-Prolla, M. Bahceci, G. Baujat, G. Baynam, E. Beckh-Arnold, A. Ben-Yehuda, M. Bhat, L. Bird, J. Bliek, A. Bottani, F. Breatnach, B. Buehler, E. Chang, C. Christenden, C. Clericuzio, I. Cordeiro, V. Culic, T. Cushing, S. Danda, M. De Roy, V. de Soberanis, A. Dieckmann, J. Dominguez, H. Dorkins, M. Doz, J. Dupont, M. Edwards, E. Crocker, F. Faravelli, T. Fiskerstrand, W. Foulkes, J. Franklin, H. Fryssira, M. Gardner, B. Gener, Y. Gillerot, H. Goel, K. Gowrishankar, A. Green, N. Gregersen, M. Hamilton, D. Horovitz, W-L. Hwu, A. Irvine, M-L. Jacquemont, S. Jagadeesh, A. Jorgensen, P. Kannu, K. Keppler-Noreuil, A. Krause, V. Krishnamurthy, A. Kumar, P. Lapunzina, N. Leonard, J. Liebelt, T. Lopponen, Dr Lozano, S.A. Lynch, S. Lyonnet, S. Maitz, T. McDevitt, G. McGillivray, A. Medeira, V. Meiner, K. Milstein, S. Nampoothiri, K. Nathanson, K. Neas, G. Neri, C. Nur Semerci, C. Ockeloen, K. Ozono, C. Panarello, J. Piard, B. Plecko, V. Puthi, W. Raith, L. Read, W. Reardon, O. Rittinger, A. Rothschild, N. Saleh, V. Saletti, H. Santos, , C. Searle, A. Selicorni, M. Simon, K. Stuurman, A. Swain, K. Szakszon, M. Teixeira, E. Thompson, F. Thonney, S. Turkmen, A. Turner, M. Van Haelst, L. Van Maldergem, C. Verellen, I.C. Verma, J. Vigneron, L. Wainwright, D. Weaver, P. Wheeler, K. White, N. Yachelevich, A. Yeung, A. Zankl.
